# A cross-sectional study of pedagogical strategies in nursing education: opportunities and constraints toward using effective pedagogy

**DOI:** 10.1186/s12909-015-0411-5

**Published:** 2015-08-25

**Authors:** Nicola Pagnucci, Franco A. Carnevale, Annamaria Bagnasco, Angela Tolotti, Lucia Cadorin, Loredana Sasso

**Affiliations:** 1 Department of Health Sciences, University of Genoa, via Pastore 1, Genoa, I-16132 Italy; 2 Ingram School of Nursing, Wilson Hall, McGill University, 3506 University Street, Montreal, Quebec H3A 2A7 Canada

**Keywords:** Nursing education, Effective pedagogy, Pedagogical strategies, Teaching taxonomy, Learning environment, Italy

## Abstract

**Background:**

The continuous, rapid evolution of medical technology, the public need for ever more complex health-care services and the stagnant global economic situation have posed difficult new challenges for the nursing profession. The need to integrate knowledge, technical skill and ethical conduct in nursing practice has become ever more evident, particularly in response to the emerging challenges over recent years. Major research studies have highlighted that high-quality responses to health needs is highly dependent on both the education received by health care professionals and the pedagogical strategies employed in such training. The aim of this study was to identify the pedagogical strategies used by teachers in nursing programs in the Italian university system and to classify them according to the didactic architectures that are used.

**Methods:**

The study sample was recruited from the entire population of nursing instructors teaching in all years of their respective programs, in every Italian university with a nursing program. A three-part questionnaire, based on a Calvani taxonomy, was designed to collect both demographic and cultural information on the sample subjects, as well as the pedagogical strategies that they may have used in their teaching practices, was administered to all nursing instructors. A five-point Likert scale was used to measure the frequency of use of different pedagogical strategies.

**Results:**

On the whole, 992 teachers participated in the study (80.1 % of the teachers contacted). Experience data suggest a highly-educated overall instructor population. The settings in which the participants carried out their teaching activities were represented mostly by large lecture halls and the number of students in their classes were for the most part rather large; over 60. Frequency of use revealed that the most commonly used method was the traditional lecture. Indeed, 85.7 % of the respondents “often” or “always” used pedagogical strategies belonging to a ‘receptive architecture’.

**Conclusions:**

Any redefining of approaches to nursing education must consider several key factors to ensure the promotion of student-focused pedagogical strategies. Only through the implementation of such pedagogical practices will it be possible to generate the knowledge and skills necessary for future professionals to be able to adequately respond to the ever more complex health care needs of the population.

## Background

In recent years the continuous, rapid evolution of medical technology, the public need for ever more complex health-care services and the stagnant global economic situation have posed complex new challenges for the nursing profession [1–3]. The rising incidence of chronic conditions, the increase in life expectancy and continuous cuts in funding for health care have led to the displacement of nursing practices to places and settings that had never before been used to provide health care [4]. Until recently, nurses and other health care professionals could always count on hospitals and clinics as stable points of reference for carrying out their profession and a population with relatively complex needs as the recipients of their care. In these current times nurses are being required to adapt to profound change. Economic and social policies of recent years [5] have led to a new distribution of patients amongst hospitals and other structures, concentrating patients with acute illnesses in hospitals and most patients with chronic conditions either at home or in community centres and clinics devoted specifically to their care [6]. The implications of this change, still ongoing in Italy, have been extensive and wide-ranging. Nurses now find themselves, on the one hand, faced ever more often with the need to provide intensive, complex care, using new technologies that are increasingly ubiquitous and indispensable in hospital settings. On the other hand, they must also care for patients with multiple chronic pathologies [7] in settings often lacking adequate safety measures [8], where they need to rely for the most part on their own skill [9] and at the same time carry out education and prevention functions; in particular, promoting the involvement of the family and others in the care of the patient [10].

The continual, acutely felt need for nurses to integrate knowledge, technical skills and ethical conduct in their practice has become ever more evident [11], particularly so in response to the challenges facing nurses over the last few years. It is only through excellence in practice, which calls for imaginative interpretation and understanding of the potentialities inherent in each individual situation, and a process of integration of the three different components of nursing practice that professionals can find effective responses to the needs for complex, intensive and challenging health care. Recent studies conducted in American [12] and European [13] health care facilities have by now amply demonstrated the strict relationship between nurses’ level of education, their numbers in health care facilities and patient mortality rates.

One of the most extensive studies conducted in recent years on nursing practices in the US [14] has produced extremely useful insight into the role of education in training future nurses. Training that manages to impart the capacity to integrate knowledge, technical skills and ethical conduct is the best way to prepare students for professional practice.

Formal separation between clinical learning and classroom learning does not foster the development of the ability to combine these three components, as is required of effective modern nursing practice. Better integrating these learning aspects during education could provide a response to the current state of fragmentation of knowledge experienced by students, and orient the nursing profession toward excellence in practice [11, 15].

Nurses work in complex, often unstructured clinical situations, where they are asked to recognize and quickly appraise and prioritize courses of action. Understanding the nature of the situation is the very basis for decision making in unpredictable and continuously changing circumstances [16, 17].

Pedagogical methods that include continuous, situated “coaching” are necessary to allow students to understand all the factors in specific clinical situations that are moreover subject to change: the importance of signs and symptoms, the patient’s, family’s and other health care workers’ requests, the resources available and any constraints present. Developing a “sense of salience” [14] calls for combining understanding and skill with the ability to put into practice the information acquired from a rich pool of knowledge. Using different modes of reasoning is essential to attaining such abilities. Indeed, nurses employ particular ways of thinking that can take into account the setting, the patients and their family and their concerns, and bring to bear a clinical imagination aimed at understanding the situation and assessing the different possibilities according to the resources and constraints present [15, 18].

This extremely multifaceted view of the professional nurse and the training that can ensure the development of the aforementioned abilities in an integrated fashion is very much in keeping with numerous international studies conducted in the field of education.

Already in 2008, in response to the challenges posed by the National League of Nursing [19], an important research study [20] highlighted that the ability to implement high-quality responses to the health needs of the population is highly dependent on the education received by health care professionals. A subsequent study [21] revealed the useful, positive effects of adopting different strategies of narrative pedagogy in student nursing programmes, with the aim of generating the capacities necessary to interpret information, analyze concepts and reflect critically on ideas or situations.

Through its Teaching and Learning Research Programme, the Economic & Social Research Council, has conducted a ten-year-long series of programmes and studies with the purpose of attaining desired learning outcomes for students in the United Kingdom. Specialized seminars, work groups, continuous discussion and exchange of information with teachers and students at an international level have brought to light some extremely important findings [22]. Such findings have been analyzed and subsequently summed up in ten “evidence-informed” principles for dissemination to a wider public.

Focusing our attention now to Italy, a review of the Italian scientific literature has revealed no trace of any research study conducted with the aim of providing an overview of the educational value of nursing degree programmes from the perspective of the didactic approaches utilized [23] and the educational outcomes attained [24, 25].

From the sparse information available in the literature regarding the introduction or use of didactic strategies of any kind whatever, it can be inferred that the educational institutions that have devoted attention to such matters are few and far between. The institutions that have documented reviews of their curricula, accompanied by the introduction or implementation of new pedagogical strategies that view students as prime actors in their own education are to date still quite rare [26]. No news at all is to be had, instead, of other didactic approaches, or “architectures”, as they have been termed, even the ‘traditional’ ones currently in use at the various Italian universities offering degree programmes in nursing.

The lack of literature data regarding the individual institutions of higher education in Italy precludes performing any analysis that may reveal the characteristics of the current educational systems adopted in nursing degree courses, in particular regarding the adoption or use of didactic strategies, as these are left up to the discretion of each institution. It is only through a thorough understanding of the current reality that valid alternatives or solutions for improvement can be formulated.

An indispensable path to acquiring such understanding is the design and implementation of a research project to delineate the pedagogical practices currently implemented in nursing programmes throughout Italy and identify any possible correlations between the experiences of teachers and students and the application of didactic architectures.

The aims of this research study are, firstly, to identify the pedagogical strategies used by teachers in nursing programmes at different educational institutions in the Italian university system, and then to map and classify them according to the didactic architectures employed. More specifically, the study has been guided by the search for answers to the following research questions.

1. What pedagogical strategies are used by teachers in the nursing programmes of various educational institutions in the Italian university system?
2. How valid is the taxonomy of pedagogical strategies proposed by Calvani as applied to nursing programmes?
3. Are there any relations between a teacher's characteristics, the learning environment and the didactic architectures employed?

## Methods

Given the lack of analogous prior studies and the potentially wide variety of pedagogical strategies adopted within a single educational institution, we have chosen to include in the study sample the entire population of regular nursing instructors teaching in all years of the program in every Italian university offering degree courses in nursing. Clinical instructors have been excluded, as their activities are expressly dedicated to teaching in laboratories or non-academic clinical settings, and hence extraneous to the aims of the study, which aims to define the pedagogical strategies used with regard to theoretical, rather than practical, aspects. Nor have substitute contract teachers been considered, due to the impossibility of consistently locating and contacting them, as they are generally not included in the teaching programmes published by the various institutions.

In order to conduct the study, it was necessary to develop a tool able to gather any and all data useful for defining the various pedagogical choices made by the collegial bodies of the individual degree courses and the teachers themselves. In order to establish what data could be considered useful for the purposes of the research, a classification of pedagogical strategies has been formulated to provide a framework for the tool itself. Given the potential size of the sampled population, it was decided to use questionnaires as the means to collect the data. This tool, which is designed to collect both demographic and cultural information on the sample subjects, as well as the pedagogical strategies they may have employed in their teaching practices, was administered to all regular nursing instructors. As for defining the various pedagogical strategies, the pedagogical section of the questionnaire was designed based on the taxonomy elaborated by Professor Antonio Calvani of the Florence University Department of Educational Sciences and Psychology [27]. The choice of this conceptual reference framework was dictated by the presumable prevalence of teacher-focused didactic strategies, a presupposition corroborated by the lack of data present in the literature regarding the use or adoption of any type of didactic strategy in Italian nursing programmes [26]. Calvani’s classification scheme is extremely clear, thanks to the formulation of hierarchical relations between macro-structures (didactic architectures) and teaching methods (pedagogical strategies), which are described in detailed and represented in tabular form (Table 1). Moreover, a considerable degree of correspondence has been found between the pedagogical strategies included in such taxonomy and those commonly adopted and cited in the relevant literature on nursing education [28–34]. Last, but not least, the choice was further supported by the possibility of using a classification formulated by an expert from Italy with knowledge of its educational and cultural system: the analysis of pedagogical strategies he performed in elaborating the taxonomy is extremely well-suited to the aims of the current research,.

**Table 1:** Hierarchical inclusion relationship between architecture and pedagogical strategies (or methods) modified by Calvani, 2012 [27]

Meta-architecture	Didactic architecture	Characteristics	Pedagogical strategy (methods)
Meta-architecture teacher focussed	Receptive (transmissive)	Control by teacher/system	Lesson
		Highly pre-structuring of information	
		Scarce or absent interaction	
	Behavioral (directive-interactive)	Control by teacher/system	Tutorial approach
			Modeling
			Field trip
		Highly pre-structuring of information	
		Extensive interaction	
		Extensive feedback control	
Meta-architecture student focussed	Situated guided discovery	Control shared by teacher and student	Problem solving
			Problem based learning
			Discussion
		Partially pre-structuring of information	
		Extensive interaction	
	Simulative	Student controlled	Simulation
			Role playing
			Case studies
		Pre-structured information or model	
		Extensive interaction with model/system	
	Collaborative	Student controlled	Cooperative learning
		More or less pre-structuring of goals	
		Extensive interaction between peer	
	Explorative	Student controlled	Project
			Brainstorming
		Little, if any, pre-structuring of information	
		Little interaction	
	Metacognitive	Teacher transfer of control to students	Self-regulated learning
		Completely student-controlled in time	

The questionnaire has been drawn up considering all the information that could be helpful in answering the research questions. To this end, it was decided to include three distinct sections in the questionnaire: the first (10 items) for information on the teachers’ demographics, education and experience; the second (3 items) on data related to the setting where the pedagogical strategies are put into practice; the third (14 items) for data on the pedagogical strategies adopted and practiced by the teacher. A five-point Likert scale (never, rarely, occasionally, frequently, always) was used to obtain answers from the respondents about strategies frequency of use.

As the questionnaire had been expressly developed for the study and was being used for the first time, it was deemed essential to subject it to a process of validation. This process included:

- a conceptual frame of reference (Calvani’s taxonomy) for content validity;
- consulting and eliciting the opinion of experts in nursing education (panel of experts) for content validity specifically for nursing;
- a preliminary test on a limited number of participants (pilot test) to evaluate its reliability following specific guidelines [35, 36];
- Cronbach’s Alpha calculation to measure internal consistency [37];
- Factor analysis to determine construct validity.

Once the target teachers were identified from the university Web sites, a database was compiled containing their names, the university and institution where they work, and their email addresses. All the educational institutions were first contacted by telephone to notify teaching coordinators of the imminent start of the study and the distribution of the questionnaires to the teachers. An introductory letter explaining the study was drafted and distributed in digital format together with the request that the questionnaires be completed.

The questionnaires were sent individually to the teachers via the Web-based “Survey-Online.com - Enuvo Gmbh - Zurich” − a Web application that enables distribution through emails containing the introductory letter, the URL of the actual questionnaire and a password to ensure security and that only one response would be received per respondent. Participants were allowed a period of 15 days to submit the completed questionnaires, which were then saved automatically by the Web application. The data saved on the application server were password protected, and thus accessible and viewable only by members of the research group. After the first 7 days, non-respondents were sent an email reminder requesting that they complete the questionnaire. Upon expiration of the 15-day data collection period, the data contained on the server were input into a database created by the Web application for subsequent statistical analysis.

The pedagogical strategies investigated in the study have been drawn from the taxonomy formulated by Calvani and adapted to nursing education. In order to analyze the didactic architectures, 7 different variables were identified and each associated to the 14 pedagogical strategies surveyed through the questionnaire. In particular, the variable of each of the architectures was calculated by averaging the Likert scale ratings of their constituent strategy variables. The meta-architectures have been studied by analyzing the average Likert scale values assigned to the variables composing each architecture (Table 1). Relations between variables were tested using the Kruskal-Wallis chi-square test and described through contingency tables.

The research protocol has been submitted and it has been approved by the Ethics Committee of the University Hospital San Martino in Genoa. Information on the teachers’ identities, telephone numbers and emails were obtained exclusively from the public-domain pages of the official Web sites of the educational institutions. The anonymity of the respondents was guaranteed by the Web application used for distributing the questionnaires and recording and saving the responses, without any possibility of tracing the identity or email address of the respondents in any way. All the teachers contacted were free to participate in the research by filling out the questionnaire online or to refuse by simply ignoring it. Submitting a completed questionnaire constituted explicit expression of consent to participate in the study. The introductory letter explained that non-participation in the research would have no influence in any way on the relationships between teacher, educational institution and university, and guaranteed the confidentiality of any information furnished.

## Results

According to the official sources of the National Federation IPASVI for the academic year 2013–2014, 221 institutions in 42 different university departments offered degree programmes in nursing. Preliminary email contact was made with all 221 institutions (100 %). The email addresses of teachers at 209 educational institutions (94.6 % of the total) were successfully obtained either from their official Web sites or by telephoning the didactic coordinators of the institutions’ nursing degree programmes. By the expiration date of the data collection period, 197 educational institutions (94.3 % of the institutions of all teachers contacted) were represented by at least one teacher who had completed the questionnaire. The number of educational institutions not represented (not even one respondent) was 12 (5.7 % of the educational institutions contacted).

The official sources of the 42 universities offering nursing degree programmes furnished the total number of teachers in their programmes; this overall population consisted of 1326 teachers. The email addresses of 1259 (94.9 % of the total population) were successfully obtained from the institutions’ Web sites. The Web application used to distribute the invitation emails to potential study participants revealed 21 email addresses that were either incorrect (17 %) or were for some reason impossible to reach (they returned a deliver error message). The overall number of teachers that successfully received the invitation email to participate was 1238 (93.4 % of the total population). Of these, 230 teachers (18.6 %) did not fill out the questionnaire (and therefore did not participate in the study), 16 (1.3 %) of those contacted began, but did not complete the questionnaire and have therefore been excluded from the study. Overall, 992 teachers participated in the study by completing the entire questionnaire (80.1 % of the teachers contacted), 694 female (70.0 %), whose average age was 48 years (s.d. = 7.49) and 298 males (30.0 %) whose mean age was 49 years (s.d. = 6.6). Of the participants, 681 (68.6 %), possessed a master’s degree. Of these, 159 (16 % of participants) attended courses of study beyond the master’s level. The participants had a mean number of 19.36 years clinical experience (s.d. = 9.14) and a mean of 11.06 years experience (s.d. = 7.91) in the field of education. Of the participants, 982 (98.5 %) were non-tenured professors and their work effort was at least partially devoted to teaching activities in 79.2 % of cases. Further details regarding the sample are shown in Table 2.

**Table 2:** Descriptive statistics of population main characteristics. No., frequency; %, the percent of answers over the total

Gender		No.	%	Age (classes)		No.	%	mean (year)	s.d
	Female	694	70.0		<30	12	1.2		
	Male	298	30.0		31–35	76	7.7		
					36–40	110	11.1		
					41–45	180	18.1		
					46–50	246	24.8		
					51–55	219	22.1		
					>56	149	15.0		
								47.32	7.62
Accademic Role		No.	%	Clinical Experience (classes)		No.	%	mean (year)	s.d
	Lecturer	982	99.0		<5	53	5.3		
	Researcher	6	0.6		6–10	152	15.3		
	Associate Professor	1	0.1		11–15	179	18.0		
	Full Professor	3	0.3		16–20	200	20.2		
					21–25	124	12.5		
					26–30	150	15.1		
					>30	134	13.5		
								19.36	9.4
Spending Time in academic activity		No.	%	Teaching Experience (classes)		No.	%	mean (year)	s.d
	part-time	786	79.2		<5	300	30.2		
	full-time	206	20.8		6–10	289	29.1		
					11–15	201	20.3		
					16–20	83	8.4		
					21–25	53	5.3		
					26–30	32	3.2		
					>30	34	3.4		
								11.06	7.91
Qualification		No.	%						
	Regional Graduation	49	4.9						
	University Diploma	11	1.1						
	Bachelor Degree	46	4.6						
	Specialization I level	164	16.5						
	Master Degree	563	56.8						
	Specialization II level	71	7.2						
	PhD	88	8.9						

The settings in which the participants carried out their teaching activities were represented mostly by large lecture halls (seating capacity of over 60 or surface area over 90 m^2^) equipped with a PC (82.1 %) and projector (91.4 %) for the teacher’s use. Moreover, the number of students in their classes were for the most part rather large, over 60 (considering the frequencies of the class-size categories: 61–80; 81–100; >100). Further details on the pedagogical settings are shown in Table 3.

**Table 3:** Descriptive statistics of learning context main characteristics. No., frequency; %, the percent of answers over the total

You teach Nursing at year:		No.	%			Facilities in learning environment		No.	%
1st	no	644	64.9			Blackboard	no	623	62.8
	yes	348	35.1				yes	369	37.2
2nd	no	585	59.0			Flip chart	no	446	45.0
	yes	407	41.0				yes	546	55.0
3rd	no	495	49.9			Teacher PC	no	178	17.9
	yes	497	50.1				yes	814	82.1
You teach nursing to a number of students of:		No.	%	mean	s.d	Projector	no	85	8.6
0–20		36	3.6				yes	907	91.4
21–40		245	24.7						
41–60		186	18.8			Multimedia whiteboard	no	898	90.5
61–80		304	30.6				yes	94	9.5
81–100		77	7.8						
>100		144	14.5			Internet and wi-fi	no	535	53.9
				3.58	1.41		yes	457	46.1
You mainly teach in classes of size:		No.	%	mean	s.d				
other		62	6.3						
small (seats ≤ 40 or area ≤ 60 m2)		118	11.9						
medium (seats 40–60 or area 60–90 m2)		299	30.1						
large (seats ≥ 60 or area ≥ 90 m2)		513	51.7						
				2.27	0.9				

Cronbach's alpha was used to measure internal consistence reliability of the questionnaire. The alpha coefficients for each subscale indicated a satisfactory degree of internal consistency as reported in Table 4. In order to check construct validity, a factor analysis was conducted on the results of the questionnaire using the “Statistical Package for Social Sciences (SPSS)” (version 21) and “Monte Carlo PCA for Parallel Analysis” software, with eigen-values >1. The results of the third section of the questionnaire in the form of 7 subscales were subjected to principal component analysis followed by Varimax rotation. This enabled extracting two factors identified as “teacher-focused meta-architecture” and “student-focused meta-architecture”. These two factors together accounted for 57 % of the variance.

**Table 4:** Internal consistency and reliability check for the seven subscales

Subscales	Cronbach Alpha
Receptive (transmissive)	0.68
Behavioral (directive- interactive)	0.71
Situated guided discovery	0.59
Simulative	0.66
Collaborative	0.68
Explorative	0.73
Metacognitive	0.69

On factor 1, teacher-focused meta-architecture, all of the subscales which should have loaded on this factor, “receptive” and “behavioral” architectures, have loadings of 0.69 or more. Another unrelated subscale, “situated guided discovery”, also has positive loadings on this factor. On factor 2, student-focused meta-architecture, four of the five relevant subscales have loadings of 0.63 or more. The factor analysis confirmed the grouping of items within the different subscales, giving support to their conceptual foundation, and show that the seven subscales maintain their place within the two meta-architectures. An analysis of the answers provided in the free text field where the respondents could indicate pedagogical strategies not included in the questionnaire list lends strong support to the validity of applying Antonio Calvani’s taxonomy to education in nursing, as hypothesized in the planning stages of the study. As anticipated by the assessments made by the panel of experts called upon to validate the classification as adapted for the field of nursing, the additional strategies cited by the respondents (i.e., showing films, case histories recounted by the patients themselves or through their diaries) can be all be referred back to an item contained in the list, which was derived from Calvani’s taxonomy, and hence classified in one of its seven didactic architectures. Such a finding indicates that all the pedagogical strategies cited by the respondents as teaching practices they used were fully represented in the classification, leading to the conclusion that application of Calvani’s taxonomy to education in nursing can indeed be deemed valid.

The results obtained with regard to pedagogical strategies, whose frequency of use was described by the respondents through a five-point Likert scale, are reported in Table 5, which reveals that the most commonly used method was the traditional lesson (mean 4.10 and s.d. = 0.73), followed by problem solving (mean 3.53 and s.d. = 1.06) and a tutorial approach (mean 3.36 and s.d. = 1.16). The questionnaire contained free text fields for specifying alternative pedagogical strategies (i.e., those not included in the questionnaire list); these elicited responses from 18 participants (1.8 %).

**Table 5:** Mean frequency of use of pedagogical strategies, didactic architectures, meta-architectures

Pedagogical strategy				Didactic architecture				Meta-architecture			
	mean	s.d	median		mean	s.d	median		mean	s.d	median
Lesson	4.10	.733	4.00	Receptive	4.10	.733	4.00	Student focussed	2.65	.867	3.00
Tutoring	3.36	1.164	4.00	Behavioral	2.88	.986	3.00				
Modeling	3.08	1.187	3.00								
Field Trip	2.08	1.233	2.00								
Problem solving	3.53	1.056	4.00	Situated guided discovery	3.20	.919	3.00	Teacher focussed	3.55	.650	4.00
Problem Based Learning	2.83	1.149	3.00								
Discussion	3.23	1.116	3.00								
Simulation	2.92	1.170	3.00	Simulative	2.82	.931	3.00				
Role playing	2.27	1.136	2.00								
Case study	3.23	1.134	3.00								
Cooperative learning	2.60	1.157	3.00	Collaborative	2.60	1.157	3.00				
Project	2.43	1.232	3.00	Explorative	2.85	1.039	3.00				
Brainstorming	2.85	1.117	3.00								
Self-regulated learning	2.09	1.185	2.00	Metacognitive	2.09	1.185	2.00				

By assigning the pedagogical strategies used by the respondents to one of the 7 didactic architectures at the uppermost hierarchical level of the taxonomy (see Table 1), it can be clearly seen that the most frequently adopted architecture is the receptive one (mean 4.10, s.d. = 0.73), while, on the contrary, the meta-cognitive architecture was the least frequent (mean 2.09, s.d. = 1.18)

The more student-focused didactic architectures, which call for shared control of the methodology between students and teacher, and moreover view the student not only as more active in the learning process, but at its very centre (guided discovery, simulative, collaborative, exploratory and meta-cognitive activities), turned out to be less frequently adopted, on average, than the teacher-focused architectures (receptive and behavioural) (see Table 5).

## Discussion

The very high participant response rate revealed the considerable interest in this topic among Italian nursing instructors. Numerous comments were received from respondents through the free-text comment boxes at the end of the questionnaire (which have not been reported here because of space restrictions), as well as by email and phone calls to the research group. Many were to express their support for the aims of the research and the urgent need to devote greater attention to pedagogical aspects of nursing education in Italy.

Information collected regarding the characteristics of the study sample demonstrates a population of highly educated teachers (in some cases, master’s and doctoral degrees), especially considering that masters-level nursing degree programs were first established in Italy only in 2001.

The fact that the sample includes a large proportion of instructors with 10 years of clinical experience or more, and with 6 to 15 years of experience in education. These background characteristics are considered favorable for the implementation of effective pedagogical methods that can stimulate imaginative interpretation and understanding in the preparation of health care professionals [14, 38].

The receptive architecture is significantly related to several variables. However, whereas 85.7 % of the respondents indicated that they “often” or “always” use pedagogical strategies belonging to the receptive architecture, the relations between the Likert scale values attributed to the receptive architecture within the individual variables studied were maintained, thereby indicating no differences in architecture use between the descriptive cases of the individual variables tested.

Significant correlations have been found between large class sizes and the frequency of use of student-focused meta-architecture pedagogical strategies (*p* = 0,0006). These highlight how the student − teacher ratio influences the possibilities for application of various pedagogical strategies (see Tables 6, 7, 8 and 9).

**Table 6:** The three most significant correlations

Correlations			*p*-value
"Meta-architecture student focussed"	vs	“Number of students (class)”	0.0006
"Meta-architecture student focussed"	vs	“Size of learning environment”	0.0012
"Meta-architecture student focussed"	vs	"Flip chart in learning environment"	0.0020

**Table 7:** The correlation between “Metarchitecture student focussed” and “number of students”

			You teach Nursing to a number of students of:						
Metarchitecture student focussed			0–20	21–40	41–60	61–80	81–100	>100	total
	never	No.	0	23	6	15	4	24	72
		row %	0.0	0.3	0.1	0.2	0.1	0.3	1.0
		column %	0.0	0.1	0.0	0.0	0.1	0.2	0.1
	rarely	No.	24	74	60	140	40	41	379
		row %	0.1	0.2	0.2	0.4	0.1	0.1	1.0
		column %	0.7	0.3	0.3	0.5	0.5	0.3	0.4
	sometimes	No.	9	96	81	106	22	63	377
		row %	0.0	0.3	0.2	0.3	0.1	0.2	1.0
		column %	0.3	0.4	0.4	0.3	0.3	0.4	0.4
	often	No.	3	50	37	38	8	16	152
		row %	0.0	0.3	0.2	0.3	0.1	0.1	1.0
		column %	0.1	0.2	0.2	0.1	0.1	0.1	0.2
	always	No.	0	2	2	5	3	0	12
		row %	0.0	0.2	0.2	0.4	0.3	0.0	1.0
		column %	0.0	0.0	0.0	0.0	0.0	0.0	0.0
	Total	No.	36	245	186	304	77	144	992
		row %	0.0	0.2	0.2	0.3	0.1	0.1	1.0
		column %	1.0	1.0	1.0	1.0	1.0	1.0	1.0

**Table 8:** The correlation between “Metarchitecture student focussed” and “classroom size”

			You mainly teach in classes of size:				
Metarchitecture student focussed			other	small	medium	large	total
				(seats ≤ 40 or area ≤ 60 m2)	(seats 40–60 or area 60–90 m2)	(seats ≥ 60 or area ≥ 90 m2)	
	never	No.	0	5	20	47	72
		row %	0.0	0.1	0.3	0.7	1.0
		column %	0.0	0.0	0.1	0.1	0.1
	rarely	No.	13	47	110	209	379
		row %	0.0	0.1	0.3	0.6	1.0
		column %	0.2	0.4	0.4	0.4	0.4
	sometimes	No.	28	48	106	195	377
		row %	0.1	0.1	0.3	0.5	1.0
		column %	0.5	0.4	0.4	0.4	0.4
	often	No.	20	16	58	58	152
		row %	0.1	0.1	0.4	0.4	1.0
		column %	0.3	0.1	0.2	0.1	0.2
	always	No.	1	2	5	4	12
		row %	0.1	0.2	0.4	0.3	1.0
		column %	0.0	0.0	0.0	0.0	0.0
	Total	No.	62	118	299	513	992
		row %	0.1	0.1	0.3	0.5	1.0
		column %	1.0	1.0	1.0	1.0	1.0

**Table 9:** The correlation between “Metarchitecture student focussed” and“Flip-chart in learning environment”

			Flip-chart in learning environment		
Metarchitecture student focussed			no	yes	total
	never	No.	37	35	72
		row %	0.5	0.5	1.0
		column %	0.1	0.1	0.1
	rarely	No.	192	187	379
		row %	0.5	0.5	1.0
		column %	0.4	0.3	0.4
	sometimes	No.	151	226	377
		row %	0.4	0.6	1.0
		column %	0.3	0.4	0.4
	often	No.	61	91	152
		row %	0.4	0.6	1.0
		column %	0.1	0.2	0.2
	always	No.	5	7	12
		row %	0.4	0.6	1.0
		column %	0.0	0.0	0.0
	Total	No.	446	546	992
		row %	0.4	0.6	1.0
		column %	1.0	1.0	1.0

Pedagogical strategies amongst the didactic architectures considered to be the most effective from a pedagogical perspective are characterized by extensive interaction between students, teachers and the institutional system. Adopting and putting into practice these strategies is strictly dependent on the student-teacher ratio, as they determine (a) the degree of support that can be offered to students, and (b) the level of student involvement in the learning process [22].

The reported learning environments consisted mainly of large-sized classrooms or lecture halls (seating capacity of over 60 or surface area over 90 m2). A significant correlation was found between classroom size and the pedagogical strategies belonging to meta- student focused meta-architectures. This helps explain the use of student-focused strategies. Settings that are conducive to promoting a contextualized climate for interaction, collaboration and synergy are essential for implementing pedagogical strategies of didactic architectures that provide for greater control by the students over the methods used and encourage them to actively participate in the learning process (student-focused).

According to the correlations revealed, the main causes of the non-adoption of pedagogical strategies belonging to the didactic architectures considered most pedagogically effective seem to stem from the excessive number of students per instructor and the large size of the environments in which teaching activities are conducted.

Regarding the teaching aids and equipment available to the teachers in carrying out their activities, a significant correlation was found between the frequency of use of strategies belonging to the student-focused architectures and the classroom presence of a flip chart. This suggests a significant influence of the use of such tools as supports for the application of pedagogical strategies that best encourage active student involvement.

Concerning the characteristics of the participating teachers, the data revealed that they possess a high degree of ‘professional maturity’ consistent with their high level of education and extensive clinical and academic experience, which would in turn imply that they possess the sensitivity and skills necessary to formulate and put into practice the pedagogical strategies deemed most effective; that is, student-focused architectures, as already demonstrated in a wide body of literature [14, 38]. However, it seems that limitations inherent in the settings where these teachers are actually called upon to practice their profession force them to adopt strategies that they deem ‘doable’; that is, teacher-focused architectures.

It appears that the current structure and organization of nursing degree programs in Italy present significant barriers to the application of effective pedagogy. Any future redefining of nursing education must consider these factors to successfully favor the adoption of student-focused pedagogical strategies. Only through implementation of such pedagogical practices will it be possible to generate the knowledge and skills necessary for future nurses to be able to adequately respond to the ever more complex health care needs of the population at large.

The demonstrated support for the validity of the Calvani taxonomy for nursing education will help promote future research and instructional development in.

A principal limitation of the study was the difficulty in obtaining data from 24 training centers that are located mainly in southern Italy, restricting the study to education centers located primarily in central and northern Italy. Further research will be needed to better examine the southern Italian context. Moreover, the study is limited by relying solely on self-report data. It is possible that there can be discrepancies between self-reported and actual pedagogical practices. Future research should examine also actual instructional practices. Finally, student learning perspectives were not included in this study. Future research should examine how student learning is related to teacher instructional practices.

## Conclusions

The results of this study reveal the high degree of interest among Italian nursing instructors in pedagogical approaches to nursing education. Barriers to implementing effective pedagogy into nursing education programs that have emerged from the study call for careful evaluation by the bodies responsible for the planning and organization of degree programs. Practicing effective pedagogy can only take place through close co-ordination of the activities of every member and institution involved at every level in the entire education process within a coherent policy framework with a principal objective to optimize learning.

Clearly, further studies will be necessary to shed light on any educational settings where specific didactic architectures have been particularly well developed and delve into the organisation of these settings, as well as the dynamics established among their various components. Only through a profound understanding of the current reality can valid alternative solutions be found for improving the reality of nursing education itself.

## Abbreviation

IPASVI: Infermieri professionali, Assistenti sanitari, Vigilatrici d’infanzia (nurses, health visitor, children’s nurses)

## References

[1] Overstreet M (2015). Transforming nursing practice with technology. Nurs Clin North Am.

[2] Elgin KH, Bergero C (2015). Technology and the Bedside Nurse: an exploration and review of implications for practice. Nurs Clin North Am.

[3] Hamer S, Cipriano P (2013). Involving nurses in developing new technology. Nurs Times.

[4] Simonazzi A (2012). Time, cash and services: Reforms for a future sustainable long-term care. Futures.

[5] Gemmill M. Research note: Chronic disease management in Europe. European Commission Directorate-General “Employment, Social Affairs and Equal Opportunities” Unit E1 - Social and Demographic Analysis, 2008.

[6] Genet N, Kroneman M, Boerma WG (2013). Explaining governmental involvement in home care across Europe: an international comparative study. Health Policy.

[7] Parekh AK, Goodman RA, Gordon C, Koh HK (2011). Managing multiple chronic conditions: a strategic framework for improving health outcomes and quality of life. Public Health Rep.

[8] Nolte E, Knai C, McKee M. Managing chronic conditions: experience in eight countries. Copenhagen, Denmark: World Health Organization on behalf of the European Observatory on Health Systems and Policies; 2008:XVII, 181.

[9] Struckmann V, Snoeijs S, Melchiorre MG, Hujala A, Rijken M, Quentin W (2014). Caring for people with multiple chronic conditions in Europe. Eurohealth Incorporating Euro Observer.

[10] Varela G, Varona L, Anderson K, Sansoni J. Alzheimer’s care at home: a focus on caregivers strain. Prof Inferm. 2011;64(2):113–7. PMC3662364 21843435

[11] Benner P. From novice to expert: Excellence and power in clinical nursing practice. London: Pearson; 2001.

[12] Aiken LH, Cimiotti JP, Sloane DM, Smith HL, Flynn L, Neff DF (2011). The effects of nurse staffing and nurse education on patient deaths in hospitals with different nurse work environments. Med Care.

[13] Aiken LH, Sloane DM, Bruyneel L, Van den Heede K, Griffiths P, Busse R (2014). Nurse staffing and education and hospital mortality in nine European countries: a retrospective observational study. Lancet.

[14] Benner P, Sutphen M, Leonard V, Day L. Educating nurses: A call for radical transformation. San Francisco: John Wiley & Sons; 2009.

[15] Benner P, Tanner C (1987). How expert nurses use intuition. Am J Nursing.

[16] Benner P (2004). Using the Dreyfus model of skill acquisition to describe and interpret skill acquisition and clinical judgment in nursing practice and education. Bull Sci Technol Soc.

[17] Benner P, Tanner CA, Chesla CA. Expertise in nursing practice: Caring, clinical judgment, and ethics. New York: Springer Publishing Company; 2009

[18] Benner P, Hooper-Kyriakidis PL, Stannard D. Clinical wisdom and interventions in critical care: A thinkingin-action approach. Philadelphia: Saunders; 1999.

[19] Author, editor. Position statement: Innovation in nursing education: a call to reform. New york: National League for Nursing Press; 2003.

[20] Greiner AC, Knebel E. Health professions education: a bridge to quality. New York: National Academies Press; 2003. 25057657

[21] Brown ST, Kirkpatrick MK, Mangum D, Avery J (2008). A review of narrative pedagogy strategies to transform traditional nursing education. J Nurs Educ.

[22] James M, Pollard A (2011). TLRP’s ten principles for effective pedagogy: Rationale, development, evidence, argument and impact. Res Papers Educ.

[23] Clark RC (2000). Four architectures of instruction. Perform Improvement.

[24] Allen MJ. Assessing general education programs. Bolton: Anker Publishing Company; 2006.

[25] Johnston-Hanson KS (2012). Nursing department education needs assessment: implementation and outcome. J Nurses Prof Dev.

[26] Sasso L, Lotti A, Guilbert JJ. Problem-Based Learning per le professioni sanitarie. Milano: McGraw-Hill; 2007.

[27] Calvani A. Per un’istruzione evidence based. Analisi teorico-metodologica internazionale sulle didattiche efficaci e inclusive. Trento: Edizioni Erickson; 2012.

[28] Löfmark A, Morberg Å, Öhlund LS, Ilicki J (2009). Supervising mentors’ lived experience on supervision in teaching, nursing and social care education. A participation-oriented phenomenological study. Higher Educ.

[29] Ariizumi Y. Action research as another literacy skill to improve academic performance: a case study of empowered language learning. Report, Lafayette College, Easton; 2006.

[30] O’Connor A, Hyde A (2005). Teaching reflection to nursing students: a qualitative study in an Irish context. Innov Educ Teach Int.

[31] Griffin MTQ, Novotny J. A Nuts and Bolts Approach to Teaching Nursing. New York: Springer Publishing Company; 2011.

[32] Horsfall J, Cleary M, Hunt GE (2012). Developing a pedagogy for nursing teaching–learning. Nurse Educ Today.

[33] Dunnington RM (2014). The nature of reality represented in high fidelity human patient simulation: philosophical perspectives and implications for nursing education. Nurs Philos.

[34] Kong L-N, Qin B, Zhou Y, Mou S, Gao H-M (2013). The effectiveness of problem-based learning on development of nursing students’ critical thinking: A systematic review and meta-analysis. Int J Nurs Stud.

[35] Iarossi G. The power of survey design: A user’s guide for managing surveys, interpreting results, and influencing respondents. Washington: World Bank Publications; 2006.

[36] Biemer PP, Lyberg LE. Introduction to survey quality. Hoboken: John Wiley & Sons; 2003

[37] Bland JM, Altman DG. Cronbach’s alpha. BMJ. 1997;314(7080):572. 10.1136/bmj.314.7080.572 PMC2126061 9055718

[38] Lave J, Wenger E. Situated learning: Legitimate peripheral participation. Cambridge: Cambridge university press; 1991.

